# Fine‐tuning fatty acid composition enhances rice resistance to bacterial blight

**DOI:** 10.1111/jipb.70314

**Published:** 2026-07-16

**Authors:** Rundong Shen, Xinbo Li, Yaqi Zhang, Liying Ding, Yongzhen Pang, Kexuan Tang

**Affiliations:** ^1^ Yazhouwan National Laboratory Sanya 572025 China

## Abstract

Genome editing to insert an inhibitory element into the 5' untranslated region of the stearoyl‐acyl carrier protein desaturase gene *OsSSI2* fine‐tuned fatty acid composition, creating mutants with enhanced bacterial blight resistance while maintaining normal growth and yield.

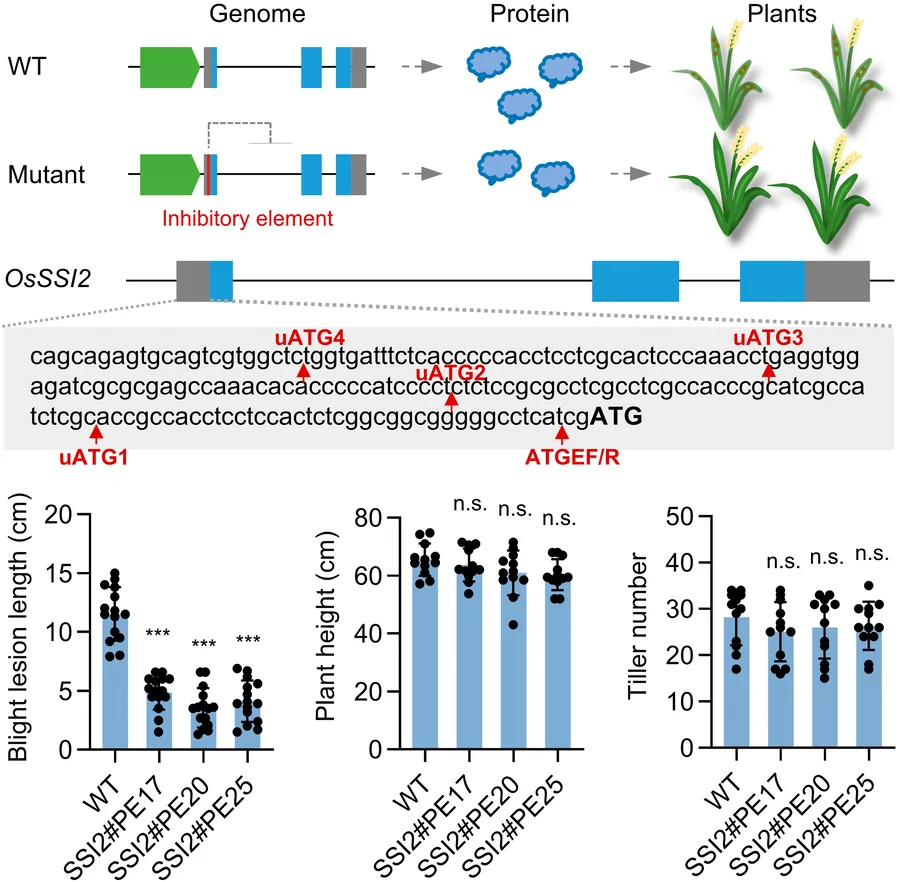

Rice (*Oryza sativa*), a staple food for over half of the global population, faces significant yield and quality losses due to diseases and pests. Among these, bacterial blight caused by *Xanthomonas oryzae* pv. *oryzae* (*Xoo*) is devastating, reducing yields by 50%–60% in Asia and Africa (Jiang et al., 2022). Resistance breeding is a cost‐effective strategy, but often encounters disease resistance–yield antagonism. For example, *OsSWEET11a/Xa13* is essential for resistance and development. Its constitutive suppression improves bacterial blight resistance, but causes pollen abortion (Wang et al., 2024b). Another key gene, *OsSSI2*, encoding stearoyl‐acyl carrier protein desaturase (SACPD), is essential for the conversion of stearic acid into oleic acid, critical for both growth and defense (Jiang et al., 2009). Complete *SSI2* knockout conferred disease resistance but caused developmental abnormalities (Quevedo‐Colmena et al., 2024). These findings underscore the potential of fine‐tuning gene expression to balance disease resistance and plant growth in crop breeding.

Promoter editing has been used to generate disease‐resistant variants without yield penalties (Oliva et al., 2019; Xu et al., 2019; Li et al., 2020; Wang et al., 2024a; Han et al., 2025). However, this approach is labor‐intensive and time‐consuming. Advances in regulatory element knock‐in provide a strategy for precisely modulating resistance gene expression to balance disease resistance and yield. Here, we inserted an upstream ATG (uATG) or a uATG‐containing element (ATGE) into the 5′UTR of *OsSSI2*, achieving fine‐tuning of gene expression (Figure 1A).

**Figure 1:**
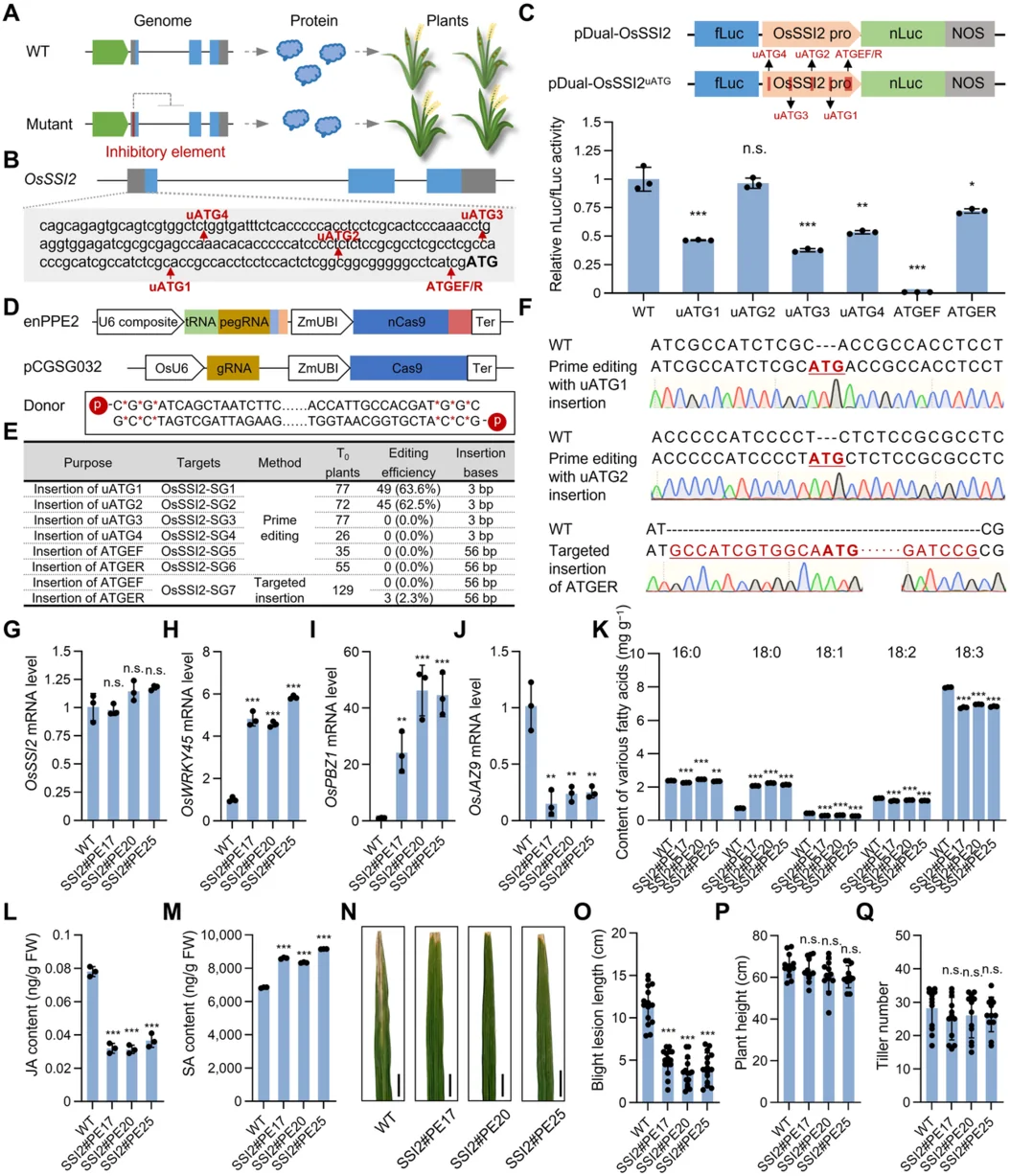
**Enhancing rice resistance to bacterial blight through** ***in‐locus*** **silencing of** ***OsSSI2*** **without compromising plant growth** **(A)** Diagram of introduction of the inhibitory element into the *OsSSI2* 5′UTR to suppress protein expression. **(B)** Insertion positions of uATG/ATGE in the *OsSSI2* 5′UTR. **(C)** Effects of the uATGs and ATGEs on nLuc/fLuc activities in dual‐luciferase assays. **(D)** Diagram of the prime‐editing vector (enPPE2), the knock‐in vector (pCBSG032), and donor ATGE. **(E)** Summary of genome editing. **(F)** Sequences of T_1_ mutants. The uATG are marked in bold red and the ATGE sequences are underlined. **(G**–**J)** mRNA levels of *OsSSI2*, *OsWRKY45*, *OsPBZ1*, and *OsJAZ9* in WT and T_1_ mutants. **(K**–**M)** Fatty acid (FA) composition and content, salicylic acid (SA) content, and jasmonic acid (JA) content in WT and T_1_ mutants. **(N)** Lesions on inoculated leaves of WT and T_1_ homozygous mutants at 14 d post‐inoculation (dpi). Bar, 2 cm. Images of WT and each mutant line were taken separately and then combined into a single figure. **(O)** Lesion lengths of WT and T_1_ mutants (*n* = 15). **(P**, **Q)** Plant height and tiller number in WT and T_1_ mutants (*n* = 12). All values are represented as means ± *SD*. ***P < 0.001; **P < 0.01; and *P < 0.05; n.s., no significant difference (two‐tailed Student's *t*‐test).

A dual‐luciferase reporter system was used to evaluate whether the inhibitory elements could suppress gene expression (Figures 1B, S1A). Four uATGs were inserted at varying positions upstream of the start codon, and a tailor‐designed ATGE (Shen et al., 2024) was inserted into the *OsSSI2* 5′UTR in both forward and reverse orientations. Dual‐luciferase assays revealed distinct functions of these elements. Four uATGs showed position‐dependent effects, with three (uATG1, uATG3, and uATG4) significantly suppressing reporter gene expression at rates of 46.5%, 37.7%, and 53.5%, respectively (Figure 1C). However, uATG2 had no effect on reporter gene expression (Figure 1C). Notably, the ATGE significantly inhibited nLuc reporter gene expression in an orientation‐dependent manner, reducing nLuc/fLuc activities to approximately 9.2% and 72.1% of the control (Figure 1C). These elements were further evaluated in stable transgenic plants.

For uATG insertion, we designed four prime editing guide RNAs (pegRNAs) and incorporated them into the prime editing vector enPPE2 (Figures 1D, S1A). These constructs were transformed into rice calli via *Agrobacterium*‐mediated transformation (Figure S2A), yielding 72, 72, 77, and 26 T_0_ transgenic seedlings. Genotyping identified 49 and 45 mutants with uATG insertions at SG1 and SG2. However, no insertions were detected at SG3 or SG4, indicating that editing efficiency is highly dependent on target sites (Figure 1E). Then, we attempted to insert the ATGE into the *OsSSI2* 5′UTR. Two pegRNAs were designed and incorporated into enPPE2. After transformation, 35 and 55 T_0_ transgenic seedlings were obtained, but no ATGE insertion was detected. Given the unsuccessful attempts, we adopted an alternative approach to insert ATGE. A single guide RNA (sgRNA) was designed and incorporated into the CRISPR‐Cas9 vector pCBSG032 (Figure 1D). The CRISPR‐Cas9 plasmid and donor DNA were co‐delivered into rice calli via biolistic‐mediated transformation, yielding 129 T_0_ transgenic seedlings (Figure S2B). Thirteen plants with ATGE insertions were identified via PCR and Sanger sequencing (Figure 1E). Representative T_1_ homozygous progenies of genome‐edited plants were subsequently collected for further analyses (Figures 1F, S3).

Quantitative RT‐PCR showed that *OsSSI2* mRNA levels in representative mutants were comparable to those in WT plants (Figures 1G, S4A), indicating that the introduced elements did not affect *OsSSI2* transcription. To determine whether the elements affect *OsSSI2* function, we examined the expression of its downstream genes, including *OsWRKY45* and *OsPBZ1*, which are important genes in the salicylic acid (SA) signaling pathway (Jiang et al., 2009). In the prime‐edited mutant lines, transcript levels of *OsWRKY45* and *OsPBZ1* were elevated 4.6‐ to 5.8‐fold and 24.2‐ to 44.6‐fold, respectively (Figure 1H, I). Similarly, the knock‐in mutant lines showed significant upregulation of *OsWRKY45* and *OsPBZ1* (Figure S4B, C), confirming that both uATG and ATGE insertions effectively influence *OsSSI2* function. Given the antagonistic interactions between the SA and jasmonic acid (JA) signaling pathways in plants, we also examined the expression of JA‐related genes and observed a marked decrease in *OsJAZ9* mRNA levels in the mutants (Figure 1J). Given that *OsSSI2* encodes SACPD, which catalyzes the conversion of stearic acid (18:0)‐ACP into oleic acid (18:1)‐ACP in fatty acid biosynthesis, we analyzed the fatty acid composition and content of rice leaves (Figure S5A). Fatty acid profiling revealed modest variations in 16:0 (palmitic acid) content, a significant increase in 18:0 (stearic acid) content, and a corresponding decrease in 18:1 (oleic acid) content in mutant plants. The contents of 18:2 (linoleic acid) and 18:3 (α‐linolenic acid) were also reduced (Figures 1K, S5B). These metabolic changes were accompanied by increased SA content and reduced JA content (Figures 1L, M, S5C), reflecting the established SA–JA antagonism.

To explore whether the observed changes in fatty acid composition and hormone content in *OsSSI2*‐edited plants contribute to enhanced bacterial blight resistance, both WT and mutant plants were inoculated with the *Xoo* strain PXO99^A^. Fourteen days post‐inoculation, lesion lengths were measured. Mutant plants showed significantly shorter lesions compared to WT plants, demonstrating enhanced resistance to bacterial blight (Figures 1N, O, S6A). No obvious morphological differences were observed between T_1_ mutants and WT plants after inoculation, with comparable plant height and tiller numbers (Figures 1P, Q, S6B, C), although individual plants of the same genotype may appear slightly different due to environmental heterogeneity (Figure S7). Since *ssi2* null mutants showed severe growth defects and yielded reductions (Jiang et al., 2009), we examined grain characteristics in the *OsSSI2*‐edited plants. The edited plants displayed no significant changes in 1,000‐grain weight, grain length, or grain width compared to WT plants (Figure S8). These findings confirm the feasibility of trait improvement through *in‐locus* gene silencing in the experimental site. Nevertheless, field trials across multiple seasons and locations are necessary to confirm the agronomic performance of these lines. Given that suppression of *OsSSI2* has been shown to enhance resistance to both bacterial blight and rice blast in previous studies (Jiang et al., 2009), the genome‐edited lines may also confer broad‐spectrum resistance. Whether these lines confer resistance to other pathogens (e.g., *Magnaporthe oryzae*) requires further investigation.

Previous studies on *SSI2* relied on null mutants or RNAi technology, which caused severe developmental penalties (Jiang et al., 2009; Quevedo‐Colmena et al., 2024). Here, we used genome editing to fine‐tune fatty acid composition, enhancing rice resistance to bacterial blight without compromising major agronomic traits. This strategy achieves precise suppression, and can be broadly applied to other genes requiring fine‐tuning.

In summary, we developed an efficient approach to enhance rice resistance to bacterial blight without compromising reproduction. This approach reduces time and labor while mitigating the trade‐offs between defense and growth. It offers a successful example of precision breeding through fine‐tuning gene expression, with the potential to overcome inherent defense–growth trade‐offs and provide technical support for developing disease‐resistant varieties in rice and other crops.

## CONFLICTS OF INTEREST

The authors declare no conflicts of interest.

## AUTHOR CONTRIBUTIONS

R.S. designed the study; R.S., X.L., Y.Z., and L.D. performed the experiments; and R.S., Y.P., and K.T. wrote and revised the manuscript. All authors have read and approved the contents of this paper.

## Supporting information

Additional Supporting Information may be found online in the supporting information tab for this article: http://onlinelibrary.wiley.com/doi/10.1111/jipb.70314/suppinfo

**Figure S1.** Schematic diagram of the dual‐luciferase reporter system and gRNA design for prime editing.
**Figure S2.** Schematic diagram of rice transformation.
**Figure S3.** Genotype of WT plants and knock‐in mutants.
**Figure S4.** The mRNA levels of *OsSSI2*, *OsWRKY45* and *OsPBZ1* in WT and T_1_ knock‐in mutants.
**Figure S5.** Effect of ATGE insertion on fatty acid composition and salicylic acid accumulation in rice.
**Figure S6.** Major agronomic traits and bacterial blight resistance of knock‐in lines.
**Figure S7.** Growth morphology of WT plants and *OsSSI2* mutants after inoculation with the *Xoo* strain PXO99^A^ for 14 days.
**Figure S8.** Comparison of grain phenotype of prime‐editing mutants (SSI2#PE17, SSI2#PE20, SSI2#PE25) with that of WT plants.
**Table S1.** Primers used in this study
