# Supplementary material for: Fine‐tuning fatty acid composition enhances rice resistance to bacterial blight
Source: J Integr Plant Biol. 2026 Jul 16;68(11):3874–6. doi: 10.1111/jipb.70314 (PMC13725603; doi:10.1111/jipb.70314)
Supplement: Supplementary file 1 [file JIPB-68-3874-s001.docx]

**Fine-tuning fatty acid composition enhances rice resistance to bacterial blight**

Rundong Shen^1^, Xinbo Li^1^, Yaqi Zhang^1^, Liying Ding^1^, Yongzhen Pang^1*^, Kexuan Tang^1*^

1, Yazhouwan National Laboratory, Sanya 572025, China

**Running title:** Tuning fatty acid composition enhances rice resistance

*Corresponding authors: Prof. Kexuan Tang tangkexuan@yzwlab.cn (K. Tang) or Prof. Yongzhen Pang pangyongzhen@yzwlab.cn (Y. Pang)

**Materials and methods**

**Plant materials and growth conditions**

The rice variety used in this study was *Nipponbare* (*Oryza sativa* L. ssp. *Japonica* cv. *Nipponbare*). Wild-type (WT) plants, transgenic plants, and genome-editing lines were grown in a growth chamber at 30°C with a 12-hour light cycle and 28°C with a 12-hour dark cycle, maintaining 70% relative humidity. Alternatively, plants were grown in paddy experimental fields.

**Vector construction**

The pDual-LUC plasmid was used as the backbone for constructing the pDual-SSI2 vector. The promoter sequence of *OsSSI2* gene was amplified and cloned into the digested pDual-LUC plasmid via Gibson assembly. Subsequently, various mutations were introduced into the pDual-LUC vector to produce pDual-SSI2^uATG1^, pDual-SSI2^uATG2^, pDual-SSI2^uATG3^, pDual-SSI2^uATG4^, pDual-SSI2^ATGEF^, and pDual-SSI2^ATGER^ vectors. For prime editing, the enPPE2 vector was digested with *Bsa*I under 37°C for 2 h. The synthesized pegRNAs were ligated into the digested enPPE2 to produce the final prime editing plasmid. For targeted insertion, the CRISPR/Cas9 plasmid was constructed as previously described (Lu et al., 2020). Briefly, the pCBSG032 vector was digested with *Bsa*I under 37°C for 2 h. The synthesized primers were annealed to form the double-stranded sgRNA and ligated into the digested pCBSG032 to produce the final CRISPR/Cas9 plasmid. Primers used in vector construction were listed in Table S1.

The luciferase reporter plasmids pDual-SSI2, pDual-SSI2^uATG1^, pDual-SSI2^uATG2^, pDual-SSI2^uATG3^, pDual-SSI2^uATG4^, pDual-SSI2^ATGEF^, and pDual-SSI2^ATGER^ were extracted using the EndoFree Mini Plasmid Kit II (Qiagen, DP118). For transient expression assay in protoplasts, 7 to 10-day-old rice green seedlings were cultured on 1/2 MS medium at 26°C under a 12 h light/12 h dark cycle. Protoplast isolation and transformation were carried out according to a previously published protocol (Zhang et al., 2011). For each experiment, an average of 5 × 10^5^ protoplasts were transformed with 10 µg of plasmid DNA. After transformation, the protoplasts were cultured in the dark at 26°C for 12 hours and harvested by centrifugation for luciferase activity measurement. Luminescence was measured with the Nano-Glo Dual-Luciferase Reporter Assay System (Promega, N1610).

**Rice transformation**

The prime editing plasmid were transformed into *Agrobacterium tumefaciens* strain EHA105 using the freezing/heat shock method. *Agrobacterium*-mediated transformation of callus cells of *Nipponbare* was conducted as described previously (Oliva et al., 2019). For targeted insertion, the CRISPR/Cas plasmid and donor DNA were delivered into rice calli through the biolistic-mediated transformation as described (Lu et al., 2020). Briefly, 0.1 pmol of prepared CRISPR/Cas9 plasmid and 2 pmol of donor DNA were mixed and added to 50 µL of Gold-Micro solution (60 mg/mL). One-month-old rice calli of *Nipponbare* were bombarded with the DNA mixture using the PDS1000/He particle bombardment system (Bio-Rad, California, USA). The transformed rice calli were cultured for plantlet regeneration. Hygromycin B (50 mg/L) was added to the culture medium to select for hygromycin-resistant calli (Figure S1).

**Genotyping**

The genomic DNA from the leaves of transgenic plants was extracted using the CTAB method. A pair of primers flanking the target site were designed for PCR to detect genome editing events. The PCR products of knock-in mutants were larger than that of the WT in DNA agarose gel electrophoresis. The PCR products were cloned into TA cloning vector pCE2-TA/Blunt-Zero (Vazyme Biotech) for Sanger sequencing. Primers used for mutant identification are shown in Table S1.

**RNA extraction and RT-qPCR**

Total RNA was extracted from the leaves of rice plants with the Spin Column Plant Total RNA Purification Kit (Sango Biotech). Reverse transcription was performed using HiScript III 1st Strand cDNA Synthesis Kit (+gDNA wiper) (Vazyme Biotech). Quantitative real-time PCR (qPCR) was performed using ChamQ SYBR Master Mix (Vazyme Biotech). The rice *Actin1* gene was used as an internal control. Three replicates were performed for each qPCR assay. Primers are shown in Table S1.

**Phenotype analysis**

WT plants and three genome-edited lines of *OsSSI2* gene were grown in an experimental field for the phenotype analysis. Plant height and tiller number were measured during harvest. Grain weight was measured based on 100 randomly chosen fully filled grains and converted to 1000-grain weight.

**Disease assays**

The *Xoo* strain PXO99^A^ was used to inoculate rice by a leaf-clipping method as described previously (Ke et al., 2017). For *Xoo* inoculation, rice plants were grown in the field until the tillering stage. *Xoo* stocks were grown on PSA medium (10 g/L tryptone, 10 g/L sucrose, 1 g/L glutamine, 12 g/L bacto-agar, pH 7.0) and incubated at 28°C for 2 days. The bacterial cells were harvested from the PSA medium and resuspended in sterile water to an OD_600_ of 0.6 for inoculation. The scissor blades were immersed in the prepared *Xoo* suspension and used to clip approximately 2 cm from the rice leaf tip. The blight lesion lengths were investigated 2 weeks after inoculation.

**Measurement of SA and JA levels**

The levels of SA and JA in the rice leaves of WT and mutant lines were quantified by Nanjing Ruiyuan Biotechnology Co., Ltd. (Nanjing, China), using ultra-performance liquid chromatography (Qsight LX 50, PerkinElmer, USA) coupled with a triple quadrupole mass spectrometer (QQQ, Qsight 420, PerkinElmer, USA). An accurately weighed sample was mixed with 10 volumes of pre-cooled chromatographic acetonitrile containing internal standard stock solution, followed by ultrasonic extraction at 4°C for 30 min and centrifugation at 12,000 rpm at 4°C for 10 min. The supernatant was collected, and the precipitate was re-extracted with 5 volumes of pre-cooled acetonitrile. The combined supernatants were mixed with appropriate amounts of C18 and GCB, vortexed for 30 s, and centrifuged at 12,000 rpm at 4°C for 10 min. The resulting supernatant was dried under nitrogen gas, reconstituted with methanol and ultrapure water, centrifuged at 12,000 rpm at 4°C for 5 min, filtered through a 0.11 μm organic phase filter membrane, and stored at −20°C until analysis.

**Fatty acid profiling**

FA analysis was performed by Nanjing Ruiyuan Biotechnology Co., Ltd. (Nanjing, China). Briefly, an amount of 0.5 g of rice leaf tissue of WT and mutant lines was ground in liquid nitrogen and mixed with 5 mL chromatographic methanol and 5 mL of chloroform for 2 hours, respectively. The extracts were centrifuged at 10,000 rpm for 10 minutes and combined to blow-dried with nitrogen gas. Then, the extracts were treated with sodium hydroxide-methanol solution and boron trifluoride-methanol solution before being mixed with hexane for gas chromatography-mass spectrometry (GC-MS).

**Statistics**

All analyses were performed using two-tailed Student’s *t*-tests to compare distributions between different groups. The *p*-value lower than 0.05 was considered to be statistically significant.

**Generative AI Usage Statement**

During the preparation of this work, the authors utilized Deepseek for language polishing. After using this tool/service, the authors carefully reviewed and revised the content where necessary, and take full responsibility for the final version of the publication.

**References**

**Ke, Y., Hui, S., and Yuan, M.** (2017). *Xanthomonas oryzae* pv. *oryzae* inoculation and growth rate on rice by leaf clipping method. Bio Protoc **7**:e2568. 10.21769/BioProtoc.2568.

**Lu, Y., Tian, Y., Shen, R., Yao, Q., Wang, M., Chen, M., Dong, J., Zhang, T., Li, F., Lei, M., et al.** (2020). Targeted, efficient sequence insertion and replacement in rice. Nat Biotechnol **38**:1402-1407. 10.1038/s41587-020-0581-5.

**Oliva, R., Ji, C., Atienza-Grande, G., Huguet-Tapia, J.C., Perez-Quintero, A., Li, T., Eom, J.S., Li, C., Nguyen, H., Liu, B., et al.** (2019). Broad-spectrum resistance to bacterial blight in rice using genome editing. Nat Biotechnol **37**:1344-1350. 10.1038/s41587-019-0267-z.

**Zhang, Y., Su, J., Duan, S., Ao, Y., Dai, J., Liu, J., Wang, P., Li, Y., Liu, B., Feng, D., et al.** (2011). A highly efficient rice green tissue protoplast system for transient gene expression and studying light/chloroplast-related processes. Plant Methods **7**:30. 10.1186/1746-4811-7-30.

**Figure S1.** Schematic diagram of the dual-luciferase reporter system and gRNA design for prime editing.

**(A)** Schematic diagram of the dual-luciferase reporter system with or without uATG/ATGE in 5’UTR of *OsSSI2*. Bases colored in red are introduced sequences of uATG or ATGE. SG indicates the single guide RNA for genome editing. The positions of SG1–SG6 were indicated by boxes. **(B)** Summary of gRNAs for prime editing.

**Figure S2.** Schematic diagram of rice transformation.

**(A)** Schematic diagram of prime editing via *Agrobacterium*-mediated transformation. The prime editing vector was introduced into rice calli through *Agrobacterium*-mediated transformation, followed by selection and regeneration to obtain transgenic rice plants. **(B)** Schematic diagram of targeted insertion via particle bombardment-mediated transformation. The CRISPR plasmids and the modified donor DNA were mixed and delivered to rice calli by particle bombardment. Subsequently, transgenic rice seedlings were obtained through selection and regeneration.

**Figure S3.** Genotype of WT plants and knock-in mutants.

**(A)** Agarose gel electrophoresis (2%) of PCR amplicons. The PCR products of the knock-in mutants are larger than that of the WT plants. **(B)** Sanger sequencing chromatograms of knock-in mutants of *OsSSI2*.

**Figure S4.** The mRNA levels of *OsSSI2*, *OsWRKY45* and *OsPBZ1* in WT and T_1_ knock-in mutants.

**(A)** The mRNA levels of *OsSSI2* in WT and T_1_ knock-in mutants. **(B)** The mRNA levels of *OsWRKY45* in WT and T_1_ knock-in mutants. **(C)** The mRNA levels of *OsPBZ1* in WT and T_1_ knock-in mutants. **(A–C)** The rice *Actin1* gene was used as an internal control for the quantitative real-time PCR (qRT-PCR). All values were represented as mean ± *SD* of three independent experiments (*n* = 3). ****P* < 0.001; ***P* < 0.01; **P* < 0.05; n.s., no significant difference (two-tailed Student’s *t*-test).

**Figure S5.** Effect of ATGE insertion on fatty acid composition and salicylic acid accumulation in rice.

**(A)** Diagram of lipid biosynthesis in the chloroplasts of plant cells. **(B)** Fatty acid (FA) composition in leaves of WT and the representative knock-in line SSI2#KI25 plants, which contained a single uORF. All values were represented as mean ± *SD* of three independent experiments (*n* = 3). ****P* < 0.001; **P* < 0.05; n.s., no significant difference (two-tailed Student’s *t*-test). **(C)** Salicylic acid (SA) content in leaves of WT and SSI2#KI25 plants. All values were represented as mean ± *SD* of three independent experiments (*n* = 3). ***P* < 0.01 (two-tailed Student’s *t*-test).

**Figure S6.** Major agronomic traits and bacterial blight resistance of knock-in lines.

**(A)** Comparison the lesion lengths of the WT plants and knock-in mutants inoculated with the *Xoo* strain PXO99^A^. Lesion length was measured 14 days after inoculation. Data were represented as mean ± *SD* (*n* = 15). ****P* < 0.001 (two-tailed Student’s *t*-test). **(B)** Plant height of WT plants and knock-in mutants. Data were represented as mean ± *SD* (*n* = 12). ****P* < 0.001; ***P* < 0.01; n.s., no significant difference (two-tailed Student’s *t*-test). **(C)** Tiller number of WT plants and knock-in mutants. Data were represented as mean ± *SD* (*n* = 12). n.s., no significant difference (two-tailed Student’s *t*-test).

**Figure S7.** Growth morphology of WT plants and *OsSSI2* mutants after inoculation with the *Xoo* strain PXO99^A^ for 14 days. Scale bar, 15 cm.

**Figure S8.** Comparison of grain phenotype of prime-editing mutants (SSI2#PE17, SSI2#PE20, SSI2#PE25) with that of WT plants.

**(A)** Morphology of grain shape in WT plants and prime-editing mutants. Scale bar, 0.5 cm. **(B)** Grain length of WT plants and prime-editing mutants. Data were represented as mean ± *SD* (*n* = 16). n.s., no significant difference (two-tailed Student’s *t*-test). **(C)** Grain width of WT plants and prime-editing mutants. Data were represented as mean ± *SD* (*n* = 16). n.s., no significant difference (two-tailed Student’s *t*-test). **(D)** 1000-grain weight of WT and prime-editing mutants. Data were represented as mean ± *SD* (*n* = 16). n.s., no significant difference (two-tailed Student’s *t*-test).

**Table S1.** Primers used in this study

| Primer Name | Primer Sequence | Application |
| --- | --- | --- |
| SSI2-KI-UP | TGTGTGGCGGCGGGGGCCTCATCGA | Construction of CRISPR/Cas9 plasmids |
| SSI2-KI-LW | AAACTCGATGAGGCCCCCGCCGCCA |  |
| SSI2-F1 | AGAGAGAGATGAGATGGCGAC | PCR for Sanger sequencing of SSI2-SG target site |
| SSI2-R1 | GTAGACCCTGACACCGCC |  |
| Donor-UP | 5`P-C*G*G*ATCAGCTAATCTTCTTGAACAGCCGCCAGCCGCTCACCATTGCCACGAT*G*G*C | Donor for targeted knock-in |
| Donor-LW | 5`P-G*C*C*ATCGTGGCAATGGTGAGCGGCTGGCGGCTGTTCAAGAAGATTAGCTGAT*C*C*G |  |
| Actin1-qF | CATTGGTGCTGAGCGTTTC | qPCR for Actin1 |
| Actin1-qR | GTACCACCACTGAGAACGATG |  |
| SSI2-qF | TGTTTGGACAAGGGCATGGA | qPCR for SSI2 |
| SSI2-qR | TGTGAGATGAAGGTCGCACG |  |
| OsPBZ1-qF | AATACGCCTAAGATGAAGAGGAATA | qPCR for PBZ1 |
| OsPBZ1-qR | ACCACAAACTTAAAGCACGAAATTA |  |
| OsWRKY45-qF | CGGGTAAAACGATCGAAAGA | qPCR for WRKY45 |
| OsWRKY45-qR | TTTCGAAAGCGGAAGAACAG |  |
| OsJAZ9-qF | GTTCTCGTCGCATTAACGGC | qPCR for JAZ9 |
| OsJAZ9-qR | CGCCGCGATAACTAGGGTAA |  |
| NLUC-WF | GCGGCGGGGGCCTCATCGATGGTCTTCACACTCGAAGA | Construction of dual-luciferase plasmids |
| NLUC-WR | GTGGGCTTGGCAATATGATGGCGTAATCATGGTCATAG |  |
| SSI2-pro-F | TCATATTGCCAAGCCCACGAA |  |
| SSI2-pro-R | ACGCCATCGATGAGGCCCCC |  |
| S-ATG1-F | GCCATCTCGCATGACCGCCACCTCCTCCACT |  |
| S-ATG1-R | AGGTGGCGGTCATGCGAGATGGCGATGCGGG |  |
| S-ATG2-F | CCCATCCCCTATGCTCTCCGCGCCTCGCCTC |  |
| S-ATG2-R | GCGCGGAGAGCATAGGGGATGGGGGTGTGTT |  |
| S-ATG3-F | TCCCAAACCTATGGAGGTGGAGATCGCGCGA |  |
| S-ATG3-R | TCTCCACCTCCATAGGTTTGGGAGTGCGAGG |  |
| S-ATG4-F | GTCGTGGCTCATGTGGTGATTTCTCACCCCC |  |
| S-ATG4-R | GAAATCACCACATGAGCCACGACTGCACTCT |  |
| S-ATGEF-F | AACAGCCGCCAGCCGCTCACCATTGCCACGATGGCCGATGGTCTTCACACTCGAA |  |
| S-ATGEF-R | CGGCTGGCGGCTGTTCAAGAAGATTAGCTGATCCGATGAGGCCCCCGCCGCCGAG |  |
| S-ATGER-F | CGGCTGGCGGCTGTTCAAGAAGATTAGCTGATCCGCGATGGTCTTCACACTCGAA |  |
| S-ATGER-R | AACAGCCGCCAGCCGCTCACCATTGCCACGATGGCATGAGGCCCCCGCCGCCGAG |  |
